# A Case of Childhood Severe Paroxysmal Cold Hemoglobinuria with Acute Renal Failure Successfully Treated with Plasma Exchange and Eculizumab

**DOI:** 10.1155/2022/3267189

**Published:** 2022-04-22

**Authors:** Jessica Pelletier, Chad Ward, Matthew Borloz, Anne Ickes, Susan Guelich, Erwood Edwards

**Affiliations:** ^1^Department of Emergency Medicine, Virginia Tech Carilion School of Medicine, Carilion Clinic, Roanoke, VA, USA; ^2^Department of Pediatric Intensive Care Medicine, Carilion Children's Hospital, Roanoke, VA, USA; ^3^Pediatric Nephrology, Virginia Tech Carilion School of Medicine, Roanoke, VA, USA; ^4^Carilion Children's Pediatric Hematology/Oncology, Carilion Clinic, Roanoke, VA, USA

## Abstract

We describe the case of a 4-year-old female who presented with sepsis and disseminated intravascular coagulation (DIC), developed ongoing intravascular hemolysis with acute renal failure from suspected pigment-induced acute tubular necrosis necessitating continuous renal replacement therapy (CRRT) for five days followed by four episodes of intermittent hemodialysis (iHD), and was subsequently diagnosed with paroxysmal cold hemoglobinuria (PCH). She was successfully treated with plasma exchange and eculizumab, a humanized monoclonal antibody targeting complement protein C5, and demonstrated significant improvement of hemolysis and recovery of renal function.

## 1. Introduction

PCH is a rare severe acute hemolytic anemia which is often triggered by viral diseases in children. We present a child with hemolytic anemia and multiorgan dysfunction, ultimately diagnosed with PCH. Diagnosis can be delayed due to its ability to mimic numerous other processes and the difficulty of obtaining confirmatory laboratory testing [1, 2]. This disease process is typically self-resolving and rarely associated with severe renal injury requiring dialysis [1–6, 7].

## 2. Case Presentation

A 4-year-old female with no significant medical history presented to the emergency department with one week of fevers, headaches, myalgias, dry cough, poor appetite, decreased urination, nausea, and vomiting. There had been no history of recent travel or sick contacts, but she was seen by her pediatrician one week prior for a well-child check and received her 4-year-old vaccines. Vital signs demonstrated oral temperature 98.2 degrees F, respiratory rate 20 breaths per minute, pulse 185 beats per minute, and oxygen saturation 95% on room air. She was noted to be ill-appearing, grunting, appeared exhausted, and was in moderate distress. On examination, she had slight pharyngeal erythema, dry mucous membranes, tachycardia, and a faint, reticular rash on her dorsal hands but no other concerning findings. Given her tachycardia and dry mucous membranes, a 20 mL/kg bolus of crystalloid was initiated and she was treated with empiric cefepime for sepsis. She received two subsequent boluses with no improvement in heart rate. Labs demonstrated leukocytosis of 45,900/μL with absolute neutrophil count 39,000/μL. The patient required multiple repeat laboratory samplings for an accurate hemoglobin level given the degree of ongoing hemolysis reported. Laboratory studies eventually demonstrated normocytic anemia with hemoglobin 10.9 g/dL and hematocrit 30.0%, normal reticulocyte count of 2.4%, normal platelet count of 302,000/μL, hyperbilirubinemia of 6.9 mg/dL, direct bilirubin 1.5 mg/dL, prerenal azotemia with blood urea nitrogen (BUN) 34 mg/dL, creatinine 0.69 mg/dL, prolonged prothrombin time (PT) of 33.6 seconds, elevated international normalized ratio (INR) of 2.9, and elevated lactate dehydrogenase (LD) of 3595 IU/L. The patient developed hypotension but responded to fluid resuscitation. The overall clinical picture at this point seemed most consistent with sepsis of uncertain etiology with associated DIC. She was transferred to the pediatric intensive care unit (PICU) at our affiliated tertiary care hospital for further management.

**Figure 1:**
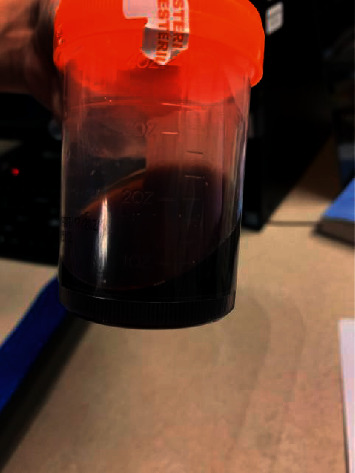
Urine sample on admission demonstrating large blood but only minimal red blood cells (25–50) per high-power field with dark brown color. This is consistent with hemoglobinuria, one of the classic presentations of childhood PCH [1, 2].

**Figure 2:**
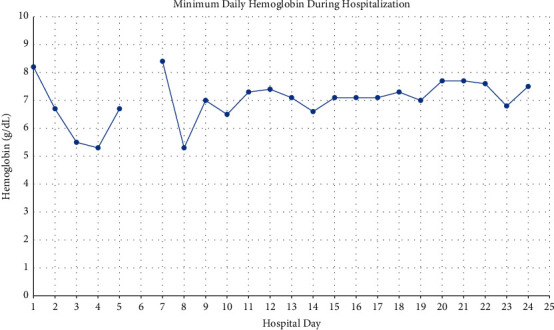
Daily minimum hemoglobin throughout hospitalization. Presenting Hgb was 10.9 g/dL, which dropped to 8.2 g/dL prior to the conclusion of hospital day 1. On hospital day 6, multiple attempts were made to obtain a hemoglobin value, but each sample was rendered invalid due to hemolysis. A spun hematocrit was measured at 13.8%.

**Figure 3:**
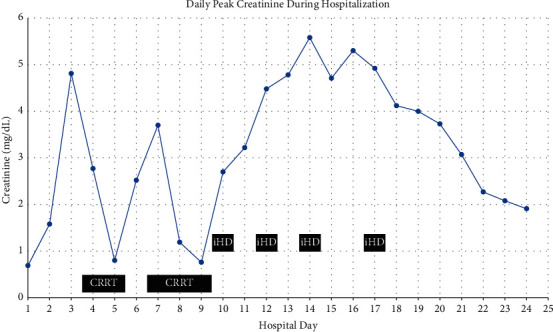
Peak creatinine trend in mg/dL throughout hospitalization. CRRT was initiated on hospital day 4, prior to the creatinine plotted for that day.

On arrival to the PICU, the patient was noted to be pale and slightly jaundiced. Haptoglobin resulted as < 20 mg/dL, consistent with hemolysis. Her fibrinogen was found to be less than 60 mg/dL and fresh frozen plasma was administered for coagulopathy. Urinalysis showed positive nitrites and leukocyte esterase but no bacteria as well as large blood with only 25–50 red blood cells (RBCs) per high-power field, and urine was dark brown in color (Figure 1). Direct antiglobulin test (DAT) showed complement positivity with negative IgG concerning for paroxysmal nocturnal hemoglobinuria (PNH) versus cold autoimmune hemolytic anemia. She was seen in consult by a hematology/oncology specialist who recommended keeping the patient and all infused products warm in the event that this was PCH. The Donath–Landsteiner (DL) antibody was sent to rule out this differential, along with testing for PNH, thrombotic thrombocytopenic purpura (TTP), and hemophagocytic lymphohistiocytosis (HLH). By hospital day 2, hemoglobin dropped to 6.7 g/dL and by hospital day 4 to a nadir of 5.3 g/dL (Figure 2). The patient developed acute kidney injury (AKI) with worsening oliguria, a BUN of 60 mg/dL, and creatinine of 1.58 mg/dL (Figure 3). Her AKI was felt to be secondary to acute tubular necrosis from hemosiderin deposition in the renal tubules in the setting of acute hemolysis (compounded by fever, ibuprofen use for fever, and dehydration). Given rapid progression of her oliguric renal failure and lack of improvement with a trial of high-dose furosemide and intravenous fluids, CRRT was initiated on hospital day 4. The patient was receiving ongoing transfusions of packed red blood cells (PRBCs) due to the degree of her hemolysis. The patient remained afebrile but was continued on empiric cefepime for sepsis in light of her ongoing leukocytosis (at this point 27,900/*μ*L). Her urine culture was negative. Azithromycin was added for coverage of *Mycoplasma* since this organism is associated with cold hemolysis. Doxycycline was added on hospital day 6 to cover for possible tickborne triggers of her illness. ADAMTS13 activity resulted as within normal limits, ruling out TTP. Infectious disease recommended testing for fungal and tickborne triggers of sepsis and hemolysis, and positron emission tomography (PET) scan to look for occult areas of infection. PNH testing with fluorescein-labeled proaerolysin (FLAER) was negative.

**Figure 4:**
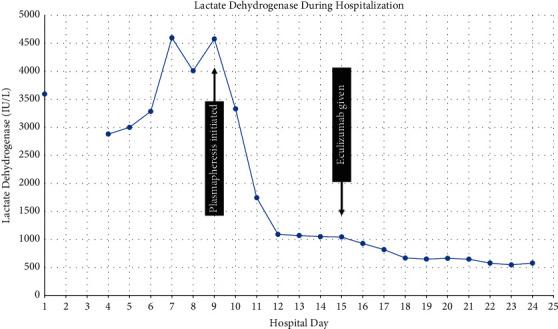
Lactate dehydrogenase (LD) trend in IU/L throughout hospitalization. The plasma exchange was initiated on hospital day 9, and eculizumab was given on hospital day 15.

Platelets dropped to a nadir of 66,000/*μ*L on hospital day 7. Testing was sent for heparin-induced thrombocytopenia and was negative, and the patient's platelet count spontaneously normalized without need for transfusion. On hospital day 9, CRRT was stopped for a trial of plasmapheresis followed by iHD. The hemolytic process significantly improved, as demonstrated by the rapid decline in lactate dehydrogenase levels (Figure 4), following initiation of plasmapheresis. The DL antibody was still in process, but empiric initiation of biologic therapy for PCH was considered. The patient received no further blood transfusions after hospital day 10. On hospital day 11, the infectious disease consultant noted that the tickborne illness panel could not be run due to the degree of the patient's hemolysis but that *Bartonella* antibody, Epstein–Barr virus polymerase chain reaction (PCR), and cytomegalovirus PCR were negative. Leukocytosis persisted at 18,400/*μ*L. PET scan revealed pneumonia, and azithromycin and doxycycline were continued for a total course of 7 days. By hospital day 14, the C4 level normalized, LD was improving, and leukocytosis resolved. Unfortunately, we were notified that the DL antibody sample was sent frozen instead of refrigerated, so it had to be recollected. HLH testing was also unable to be run, but the ferritin level was not excessively elevated at 2566 ng/mL, making this diagnosis less likely.

The patient had significant improvement following therapeutic plasmapheresis, using a protocol standard for cold agglutinin disease. She had daily 1*x* volume exchanges with fresh frozen plasma replacement for three consecutive days. Care was taken that her blood be warmed to 87 degrees to avoid exacerbating hemolysis. The patient had received eleven transfusions of PRBCs up to this point in the admission. She was then given a single dose of eculizumab 600 mg (patient weight was 21.7 kg at the time) along with steroids on hospital day 15 for empiric treatment of presumed PCH. Eculizumab was chosen over rituximab because she had demonstrated a complement-consuming process and after a shared-decision-making conversation with the family. The DL antibody test resulted positive on hospital day 18, confirming the diagnosis of PCH. She was transferred to the pediatric floor with improving urine output and significant reduction in hemolysis (last LD 545 IU/L) with no further PRBC transfusion requirements (Figure 4). The patient was discharged on hospital day 24 with outpatient hematology/oncology and nephrology follow up. Her renal function significantly improved from a creatinine peak that exceeded 5 mg/dL to a level of 2 mg/dL at discharge, with no ongoing requirements for dialysis (Figure 3).

## 3. Discussion

PCH is an intravascular hemolytic anemia mediated by inappropriate activation of the complement pathway. An IgG antibody attaches to the P antigen on RBCs at cool temperatures (for example, in acral areas) and when heated (when the blood returns to the core) leads to complement-mediated RBC lysis [5]. PCH is therefore known as a “biphasic” hemolytic anemia [1, 3–6, 8]. PCH most commonly occurs in male children (male:female ratio 2.1 : 1) around age 4–5 and follows a viral illness in 70% of cases [1, 4–6, 9]. Common viral infections implicated in triggering PCH include Epstein–Barr virus, cytomegalovirus, coxsackievirus, adenovirus, parvovirus, measles, mumps, varicella, or influenza [3–5]. Bacterial infections associated with PCH include *Mycoplasma pneumoniae* and *Haemophilus influenzae* [3, 5]. PCH has also been known to follow childhood vaccination [6]. The P antigen on RBCs is the binding site for parvovirus on RBC membranes, so it is hypothesized that molecular mimicry may play a role in causing this autoimmune phenomenon [5, 6]. Our patient's family described receipt of her childhood vaccinations (measles, mumps, and rubella; polio; diphtheria-tetanus-pertussis; and varicella) one week prior to onset of her symptoms, and she was found to have pneumonia on PET scan. Either process may have set off the course of her PCH.

The classic presentation of childhood PCH consists of pallor, jaundice, fevers, and hemoglobinuria [4, 5]. Other symptoms can include abdominal pain, nausea, anorexia, myalgias, cough, confusion, and fatigue [2–4]. Our patient exhibited nearly all of these symptoms on initial presentation. Hemoglobin typically drops below 6 g/dL (as ours did by day 4 of admission) and laboratory findings are consistent with hemolysis, including low haptoglobin, elevated LD, elevated indirect bilirubin, and urinary hyperbilirubinemia [3, 4, 9]. Patients can experience a relative reticulocytopenia in the first 2–4 days, thought to be due to a lag in reticulocyte production and possibly targeted destruction of reticulocytes bearing the P antigen [2–6, 9]. Blood smear findings in PCH can be variable and include spherocytosis, anisocytosis, erythrophagocytosis (classic for PCH but infrequently seen), neutrophil erythrocyte rosettes, fragmented RBCs, and RBC agglutination (though less common than in cold agglutinin disease) [3, 5, 7, 8]. Our patient exhibited normochromic, normocytic RBCs with anisocytosis on her initial peripheral smear, and spherocytes were noted on day 5.

The direct antiglobulin test (DAT), also known as direct Coombs test, can be used to identify binding of complement, antibodies, or both to the surface of RBCs. This is usually positive for binding of C3 but can be negative for C3 binding, negative for IgG, or both in PCH [1, 2, 6]. Our patient had a positive DAT on hospital day 1 and was negative on retesting after hospital discharge. The confirmatory test, the DL test, involves incubating the patient's blood at 4 degrees Celsius to induce binding of IgG to the P antigen, then rewarming the blood to 37 degrees Celsius to induce hemolysis. A control sample is kept at 37 degrees Celsius without cooling. The test is highly specific but not sensitive due to the possibility for patient serum to have variable amounts of complement present [1–6, 9]. Severe cases of PCH have been associated with acute renal failure, likely secondary to the intravascular hemolysis, but this is rare [1, 4, 5]. Given her requirement for renal replacement therapy, our patient fell into the severe category of PCH.

Most cases of childhood PCH are self-limited and are treated supportively by keeping the patient warm (to prevent hemolysis) and transfusing as needed for anemia [1–6]. Recurrence is fairly uncommon but when it does occur, it usually takes place within the first 21 months of the first episode [2]. There is limited evidence to support the efficacy of corticosteroids for the treatment of PCH [1, 3–6]. The first-line treatment for severe, refractory cases is rituximab, a monoclonal antibody targeting CD20 [1, 3]. Other potential treatment options include intravenous immune globulin (IVIG) and azathioprine [6]. Eculizumab, a terminal complement inhibitor, has been a controversial therapy with minimal supporting evidence up to this point [2, 3, 6]. One case report in an adult with PCH demonstrated no efficacy, but it is difficult to know whether this is applicable since the pathophysiology of PCH in adults is inherently different. Adult PCH is a rare disease but was previously seen as a secondary process in patients with tertiary syphilis or malignancies [1, 3, 10]. In a randomized controlled trial of 87 adult patients with PCH who underwent treatment with either eculizumab or placebo, the eculizumab group had significant reductions in hemolysis and blood product administration and improvement in quality of life metrics [11]. Another case report describes the efficacy of a single dose of eculizumab to induce clinical improvement in a corticosteroid-refractory case of PCH in a 4-year-old boy [9].

Given the severity of our patient's case of PCH, we reached out to other institutions that see higher volumes of patients with this illness to determine whether they typically turn to steroids, eculizumab, or rituximab as a first-line agent in cases that do not self-resolve. Since there were delays to obtaining the confirmatory DL test for PCH, it was not exactly clear whether we should utilize rituximab, the first-line agent for PCH. We discussed the risks and benefits of all options and the family preferred a combination of steroids and eculizumab over rituximab due to the side effect profile of rituximab. Therapy with plasmapheresis significantly reduced the acute hemolytic process in this patient while in the ICU. This decreased the amount of blood product being administered. The addition of eculizumab further reduced the hemolytic process allowing for further renal recovery without the need for renal replacement therapy at discharge.

To our knowledge, we describe here the second case of severe childhood PCH successfully treated with eculizumab [9]. The previously described case also involved a 4-year-old child, and steroids were trialed without improvement of his severe hemolytic anemia (with a nadir of 3.8 g/dL) [9]. He developed shock and altered mental status secondary to the severity of his anemia, but did not progress to renal failure as seen in our patient requiring dialysis. Ultimately eculizumab was started on hospital day 4, leading to resolution of his hemolysis and no further need for transfusion [9]. As stated previously, most cases of PCH are fairly mild and self-resolve. Our patient was refractory to standard supportive care measures, demonstrating ongoing hemolysis and transfusion requirements as well as acute renal failure necessitating CRRT. Her need for transfusions and dialysis ceased after acute stabilization with plasmapheresis and the hemolytic process further improved with complement inhibition with eculizumab.

## 4. Conclusion

We describe a child with severe hemolytic anemia and renal failure requiring two weeks of renal replacement therapy due to PCH. PCH is usually a self-resolving process, but in life-threatening courses such as that of our patient, plasmapheresis, steroids, and eculizumab should be considered.

## Data Availability

All the data generated or analyzed during this study are included in this published article.
